# Characterization of Gut Microbiota in Patients with Active Spreading Vitiligo Based on Whole-Genome Shotgun Sequencing

**DOI:** 10.3390/ijms26072939

**Published:** 2025-03-24

**Authors:** Hyun Jeong Ju, Woo Hyun Song, Ji Hae Shin, Ji Hae Lee, Jung Min Bae, Young Bok Lee, Minho Lee

**Affiliations:** 1 Department of Dermatology, St. Vincent’s Hospital, College of Medicine, The Catholic University of Korea, Seoul 16247, Republic of Korea; hyd0116@naver.com (H.J.J.); shinjihae1001@gmail.com (J.H.S.); l.jihaemd@gmail.com (J.H.L.); jminbae@gmail.com (J.M.B.); 2 Department of Life Science, Dongguk University-Seoul, Goyang 10326, Republic of Korea; dngus13579@gmail.com; 3 Department of Dermatology, Uijeongbu St. Mary’s Hospital, The Catholic University of Korea, Seoul 11765, Republic of Korea

**Keywords:** autoimmune, dysbiosis, gut microbiome, metabolic pathways, shotgun sequencing, vitiligo

## Abstract

Vitiligo is an autoimmune skin disease with a significant psychological burden and complex pathogenesis. While genetic factors contribute approximately 30% to its development, recent evidence suggests a crucial role of the gut microbiome in autoimmune diseases. This study investigated differences in gut microbiome composition and metabolic pathways between active spreading vitiligo patients and healthy controls using shotgun whole-genome sequencing in a Korean cohort. Taxonomic profiling reveals distinct characteristics in microbial community structure, with vitiligo patients showing an imbalanced proportion dominated by Actinomycetota and Bacteroidota. The vitiligo group exhibited significantly reduced abundance of specific species including *Faecalibacterium prausnitzii, Faecalibacteriumduncaniae*, and *Meamonas funiformis*, and increased *Bifidobacterium bifidum* compared to healthy controls. Metabolic pathway analysis identified significant enrichment in O-glycan biosynthesis pathways in vitiligo patients, while healthy controls showed enrichment in riboflavin metabolism and bacterial chemotaxis pathways. These findings provide new insights into the gut–skin axis in vitiligo pathogenesis and suggest potential therapeutic targets through microbiota modulation.

## 1. Introduction

Vitiligo is an autoimmune skin disease caused by destruction of melanocytes, with a worldwide prevalence of 0.5–1% [1]. It has a tremendous psychological burden especially in areas with skin of color and it is associated with a higher risk of developing various other autoimmune diseases such as thyroid disease, inflammatory bowel disease, and rheumatoid arthritis [2]. While there is a clear genetic predisposition to the disease, it is thought that genetics only contribute about 30% of the pathogenesis [3], with other mechanisms including environmental factors and altered inflammatory and immune responses. Therefore, gut microbiome is a subject of research to explore pathogenesis of various autoimmune diseases including metabolic, neurologic, autoimmune rheumatic, and gastrointestinal diseases.

Recent evidence suggests that human microbiome interacts with inflammatory and regulatory T cells (Tregs) to influence the progression and exacerbation of a variety of autoimmune diseases including rheumatoid arthritis, systemic lupus erythematosus, and inflammatory bowel disease [4]. Gut microbes, or their secreted metabolites, directly interact with gut-associated lymphoid tissues, leading to immune cell differentiation [5]. Moreover, they also modulate host genes through microRNAs, short non-coding RNA sequences that silence gene expression [5]. Affected host genes include those linked to differentiation of T cells, production of interleukins, proliferation of intestinal epithelial cells, gut permeability, and autophagy process [6].

Several studies have shown that patients with vitiligo exhibit differences in their gut microbiome compared to healthy controls using traditional 16s rRNA gene sequencing method. Specifically, individuals with vitiligo showed reduced alpha diversity in gut microbiome, with lower levels of *Staphylococcal thermophiles* and higher levels of *Bacteroides fragilis* [7]. Microbial composition of vitiligo lesional skin was also different compared to non-lesional sites characterized by reduction in protective bacteria like *Bifidobacterium* and enrichment in other bacteria [8,9]. Treatment with oral and topical antibiotics induced a redistribution of gut T cells and accumulation of skin Tregs in a mouse model [10]. Increasing evidence indicates that skin and gut dysbiosis is linked to induction of innate immunity and stress markers in patients with vitiligo.

While some patients show lifelong stability or steady progression of few vitiligo lesions, others have a rapid progression throughout the entire body surface area [11]. The cause of this rapid progression or active autoimmune reaction is not clearly understood. Therefore, we aimed to investigate differences in the composition and metabolic pathways of the gut microbiome in active spreading vitiligo patients compared with healthy controls in a Korean cohort by employing shotgun whole-genome sequencing. While shotgun and 16S sequencing provide two different channels to understand microbial composition, shotgun sequencing allows for a more detailed and comprehensive detection of all genomic DNA present in a sample including bacteria, fungi, viruses, and archaea [12]. Through taxonomic and metabolic pathway analyses using shotgun sequencing, we can achieve a more complete understanding of the role of microbial composition and function in the active progression of vitiligo.

## 2. Results

### 2.1. Clinical Characteristics

A total of 10 generalized vitiligo patients with active spreading state were enrolled in this study. Active spreading was defined as the presence of either hypochromic lesions or confetti lesions with appearance of new lesion during the last 3 months. Fecal samples were collected, and 10% were taking probiotics. The mean age of vitiligo patients was 36.9 and 8 females were included. The mean Vitiligo Area Scoring Index (VASI) was 30.1 and mean duration was 12.6 years. Two patients had thyroid disease, and one patient had diabetes. Probiotic use was reported in 10% and supplementary vitamins in 60%.

### 2.2. Alpha and Beta Diversity Assessment

**Figure 1:**
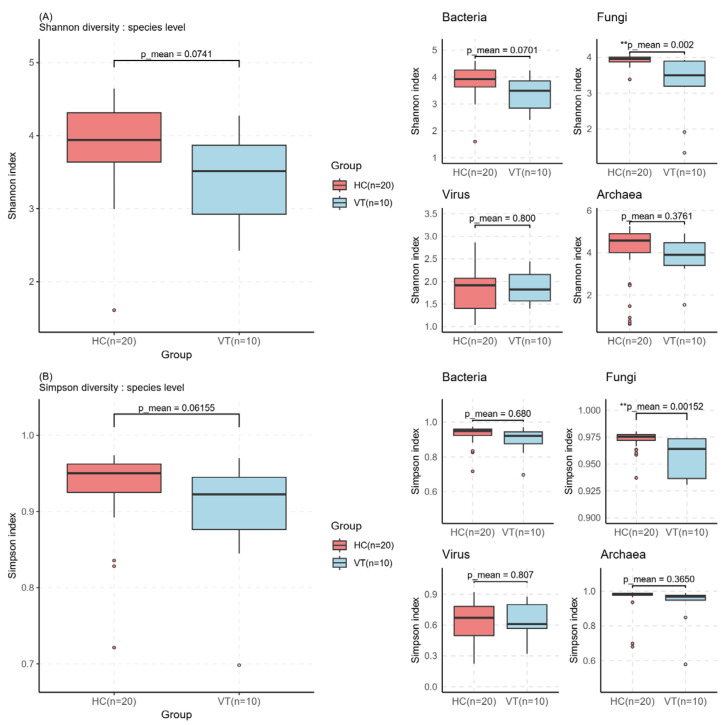
The α-diversity analysis of gut microbiota in vitiligo (VT) and health control (HC) groups at species level using (**A**) Shannon’s diversity, (**B**) Simpson’s diversity. ** *p* value < 0.01.

Alpha diversity between the vitiligo patients (VT) and healthy control (HC) groups at species level was calculated using the Simpson index and Shannon index (Figure 1). The VT group exhibited lower Simpson index and Shannon index compared with the HC group, although the difference was not statistically significant. This trend was consistently observed at the genus and species levels. Fungus showed the most significant difference between the two groups. For viruses and archaea, the diversity differences were relatively minimal.

**Figure 2:**
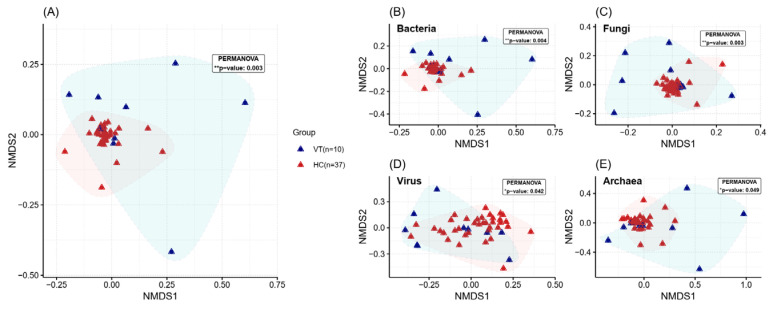
The β-diversity analysis of gut microbiome in vitiligo (VT, blue) and health control (HC, red) groups using NMDS plot across multiple microbial domains: (**A**) Overall microbial community, (**B**) Bacteria, (**C**) Fungi, (**D**) Virus, and (**E**) Archea. * *p* value < 0.05, ** *p* value < 0.01.

Beta diversity was analyzed using the Bray–Curtis method to calculate distances based on species abundance differences between samples. While the individual values of the HC group were closely clustered together showing similar distribution, the VT group showed distinct distribution compared with the HC group with larger variation between samples (*p* value, 0.005). This pattern suggests that the gut microbiota of the VT group was imbalanced, in contrast to the consistent homogeneity observed in the HC group. This pattern appeared similar throughout the microbial domains (Figure 2). On the other hand, virus showed a large variation among each sample in both groups, which could be because the gut virome is highly individualized with significant interpersonal variation but low intrapersonal diversity over time [13,14].

Overall, these findings indicate that the VT group has an imbalanced microbiome, with lower diversity and greater variability in microbial composition compared to the HC group. This pattern suggests that the microbiome in the VT group is less resilient and may lack the stability observed in the HC group.

### 2.3. Taxonomy Analysis

**Figure 3:**
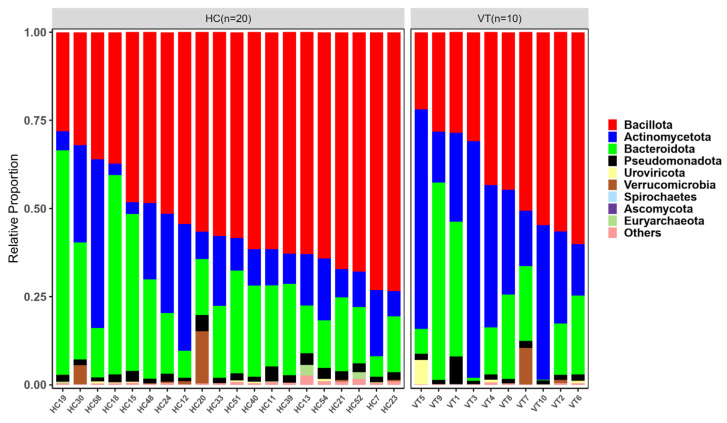
The gut microbial composition of all domains at the phylum level between the vitiligo (VT) and healthy control (HC) groups.

Taxonomic profiling reveals a distinct characteristic in microbial community structure in the VT group compared with the HC group. The three major bacterial phyla were *Bacillota*, *Actinomycetota*, and *Bacteroidota* in both groups. However, the VT group showed an imbalanced proportion of phyla dominated by *Actinomycetota* and *Bacteroiota*, although statistically not significant (Figure 3).

**Figure 4:**
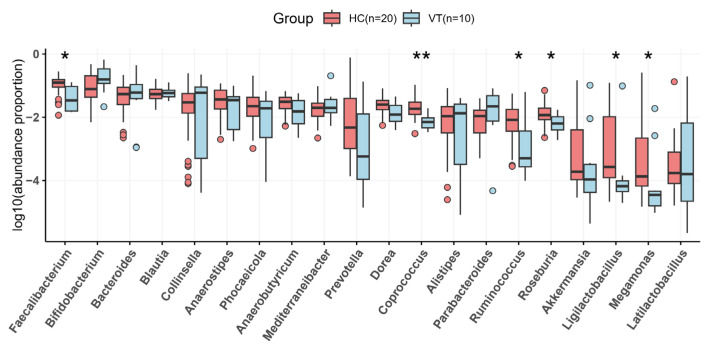
The gut microbial abundance of the vitiligo (VT) and healthy control (HC) groups at the genus level. * *p* value < 0.05, ** *p* value < 0.01.

**Figure 5:**
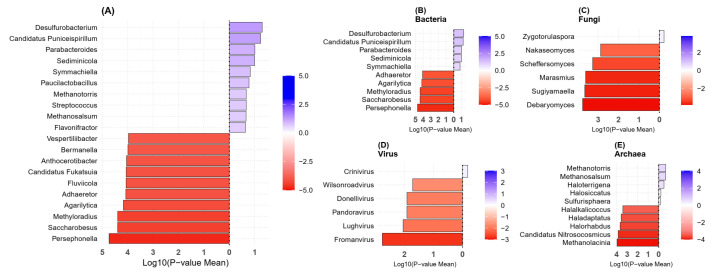
The most significantly enriched genus between the vitiligo (VT, blue) and healthy control (HC, red) groups across multiple microbial domains: (**A**) Overall microbial community, (**B**) Bacteria, (**C**) Fungi, (**D**) Virus, and (**E**) Archaea. The color scale represents the log-transformed mean *p* value, indicating the statistical significance of each pathway and the lower *p* values are indicated in darker color gradient.

The bacterial diversity was found to be reduced in patients with vitiligo at the genus level. *Faecalibacterium*, *Coprococcus*, *Ruminococcus*, *Roseburia*, *Ligilactobacillus*, and *Megamonas* were reduced compared to the HC group, while *Parabacteroides* and *Bifidobacterium* were slightly enriched in the VT group (Figure 4). A variety of genus that showed significant differences, although with very low quantity, were distributed in HC groups (Figure 5). Although their roles are not well known, they are thought to be members that each have unique characteristics, and all contribute to the diversity of the microbial ecosystem.

**Figure 6:**
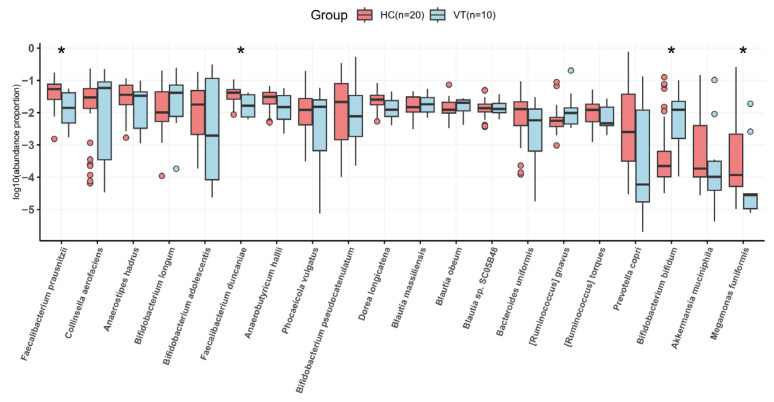
The gut microbial abundance of the vitiligo (VT) and healthy control (HC) groups at the species level. * *p* value < 0.05.

**Figure 7:**
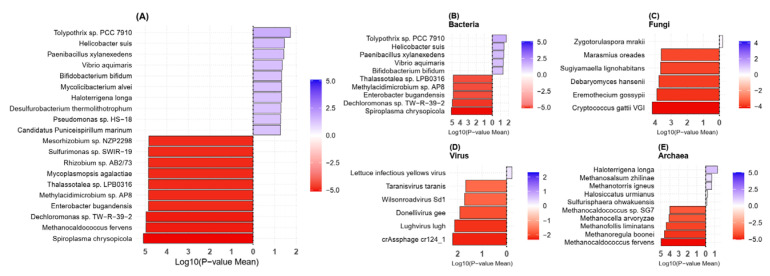
The most significantly enriched species between the vitiligo (VT, blue) and healthy control (HC, red) groups across multiple microbial domains: (**A**) Overall microbial community, (**B**) Bacteria, (**C**) Fungi, (**D**) Virus, and (**E**) Archaea. The color scale represents the log-transformed mean *p* value, indicating the statistical significance of each pathway and the lower *p* values are indicated in darker color gradient.

Species-level analysis according to the most abundant species shows that *Feacalibacterium prausnizii*, *Feacalibacterium duncanieae*, and *Megamonas funiformis* were reduced and *Bifidobacterium bifidum* was enriched in the VT group compared to the HC group (Figure 6). The HC group were enriched by a wide variety of species, including *Spiroplasma chrysopicola* (*p* value = 0.000008), *Dechloromonas* sp. (*p* value = 0.000011), and *Enterobacter bugandensis* (*p* value = 0.000014) (Figure 7).

Among fungi, *Zygotorulaspora mrakii* was significantly enriched in vitiligo patients, while five species including *Cryptococcus gattii* VGI and *Eremothecium gossypii* showed significant depletion. For viruses, *Lettuce infectious yellows virus* showed enrichment in vitiligo, while *crAssphage cr124_1* and *Lughvirus lugh* were depleted. Three archaeal species—*Haloterrigena longa*, *Methanosalsum zhilinae*, and *Methanotorris igneus*—showed positive enrichment in vitiligo patients while several species including *Methanocaldococcus fervens, Methanoregula boonei, Methanofollis liminatans, Methanocella arvoryzae*, and *Methanocaldococcus* sp. SG7 showed significant depletion in vitiligo patients (Figure 7).

### 2.4. Metabolic Pathway Enrichment Analysis

The comparative pathway analysis revealed distinct molecular signatures between vitiligo and healthy control groups. The vitiligo group showed significant enrichment in several key metabolic and biosynthetic pathways. Most notably, O-glycan biosynthesis pathways (both other types and mannose types) showed the highest enrichment (*p* value = 0.000155 and *p* value = 0.000639, respectively). Other significantly enriched pathways in the vitiligo group included terpenoid backbone biosynthesis (*p* value = 0.006250), RNA polymerase activity (*p* value = 0.007424), and lipoic acid metabolism (*p* value = 0.009682). Additionally, endocrine resistance (*p* value = 0.012151), spliceosome function (*p* value = 0.016534), and photosynthesis-related pathways (*p* value = 0.026934) were upregulated in vitiligo patients.

**Figure 8:**
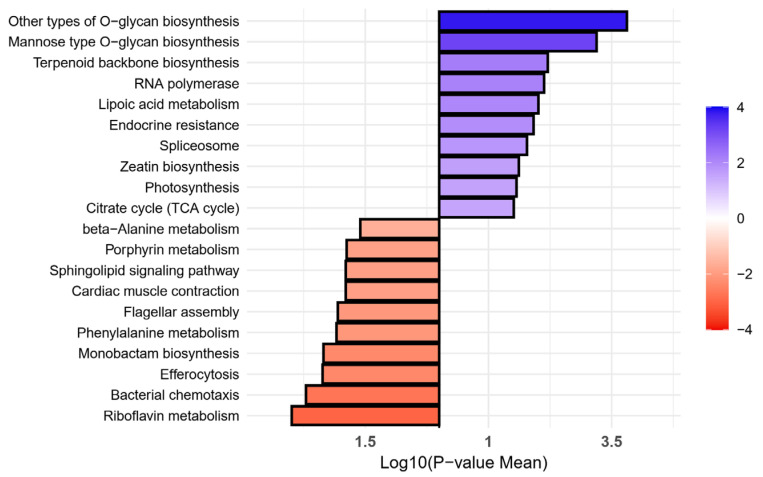
The pathway analysis of the vitiligo (VT, blue) and health control (HC, red) groups. The color scale represents the log-transformed mean *p* value, indicating the statistical significance of each pathway and the lower *p* values are indicated in darker color gradient.

Conversely, the healthy control group showed enrichment in different pathways, with riboflavin metabolism showing the strongest association (*p* value = 0.001018), followed by bacterial chemotaxis (*p* value = 0.001982) and efferocytosis (*p* value = 0.004303). Notable enrichments were also observed in monobactam biosynthesis (*p* value = 0.004485), phenylalanine metabolism (*p* value = 0.008238), and cardiac muscle contraction (*p* value = 0.012696) (Figure 8).

## 3. Discussion

Our study examined the fact that the Korean vitiligo patients exhibited reduced gut microbial diversity and greater variability in microbial composition compared to the healthy control. The bacterial community in both groups primarily consisted of Bacillota, Actinomycetota, and Bacteroidota phyla, though vitiligo patients showed an imbalanced proportion dominated by Actinomycetota and Bacteroidota. At the species level, *Feacalibacterium prausnizii*, *Feacalibacterium duncanieae*, and *Megamonas funiformis* were reduced and *Bifidobacterium bifidum* was enriched in vitiligo patients. Among non-bacterial microbes, *Zygotorulaspora mrakii* (fungi), *Lettuce infectious yellows virus*, and several archaeal species were significantly enriched in vitiligo patients. Metabolic pathway analysis reveals vitiligo patients had significant enrichment in O-glycan biosynthesis. Overall, the findings indicate that vitiligo patients have a less resilient, less diverse, and more variable gut microbiome compared to healthy controls, suggesting microbiome dysbiosis may play a role in the disease.

Recent research highlights the significant role of epigenetic modifications triggered by environmental factors in autoimmune diseases. The gut microbiome can play a pivotal role in modulating epigenetic modifications that can influence the development and progression of autoimmune diseases [15]. Disruptions in the normal microbial community can alter the production of key metabolites like short-chain fatty acids, particularly butyrate, which functions as a histone deacetylase inhibitor and affects gene expression in immune cells [15,16]. Altered tryptophan metabolism has been implicated in autoimmune pathogenesis [17]. These changes can affect the innate immune response via the level of toll-like receptors of antigen-presenting cells and the adaptive immune response by regulating Th17/Treg balance and Treg differentiation [18,19]. Another mechanism by which gut microbiome alterations contribute to autoimmune disorders is through molecular mimicry, where the immune system attacks the body’s own tissues due to structural similarities between self-antigens and microbial components, triggering autoimmune responses [14,20]. Research has shown that restoration of a healthy gut microbiome through dietary intervention, probiotics, or fecal microbiota transplantation can help reverse some of these epigenetic modifications.

Several studies have investigated microbial dysbiosis in vitiligo pathogenesis. Ganju et al. demonstrated reduced alpha diversity in the gut microbiome of vitiligo patients [9]. Subsequent research by a European group focused on skin microbiome changes, revealing distinct dysbiosis patterns in vitiligo lesions compared to non-lesional sites [8]. Their study showed reduction in Bacteroides and Bifidobacterium and increased mitochondrial DNA in the deep layer of lesional skin. A Chinese group revealed that levels of 23 serum metabolites including taurochenodeoxycholate and L-NG-monomethyl-arginine were different in vitiligo patients from healthy control and showed correlation with microbial changes [21].

The latest investigations using advanced shotgun sequencing have provided deeper insights. Luan et al. observed decreased alpha diversity, with decreased *Staphylococcus thermophiles* and increased *Bacteroides fragilis* in patients with vitiligo [7]. They also identified enrichment of NOD-like receptor signaling pathways and alterations in metabolic molecules related to cysteine and galactose degradation. Comprehensive meta-analysis by Wang et al. synthesized data from multiple studies, confirming consistent patterns of reduced microbial diversity and altered metabolic pathways across different populations with vitiligo [22]. These findings collectively suggest that microbiome dysbiosis may play a crucial role in vitiligo pathogenesis and could represent a potential therapeutic target.

Our current study took a deeper look into gut dysbiosis of active spreading vitiligo patients compared with age, sex-matched Korean healthy population employing shotgun sequencing method. As expected, the taxonomic analysis revealed distinctive characteristics of gut microbiota in vitiligo patients. The reduction in alpha diversity across multiple microbial kingdoms, particularly pronounced in fungal species, suggests a broader disruption of the microbial ecosystem. This reduced diversity could compromise the gut barrier function and immune regulation, potentially creating an environment conducive to autoimmune responses. The predominance of phyla Actinomycetota and Bacteroidota coupled with reduced microbial diversity indicates a potential shift from a healthy balanced microbiome to an imbalanced and potentially pro-inflammatory state. In a meta-analysis, gut microbial changes in the phyla Bacteroidota and Actinomycetota have been implicated in multiple autoimmune conditions [23].

In species level, vitiligo patients showed significantly reduced *Faecalibacterium prausnitzii*, *Faecalibacterium duncaniae*, and *Meganonas funiformis* and increased *Bifidobacteirum bifidum*. *Faecalibacterium prausnitzii* and *Faecalibacterium duncanieae* abundance have been reported to be decreased in inflammatory bowel diseases [24,25]. *Faecalibacterium prasuntzii*, which was also decreased in our vitiligo patients, exerts anti-inflammatory activity by inducing Treg cells through butyrate production. *Faecalibacterium duncanieae* has beneficial effects owing to its production of butyrate and other short-chain fatty acids, which are important for colonic epithelial cell metabolism and protection against colonic diseases [26,27]. Interestingly, *Bifidobacterium bifidum*, which is often considered a probiotic, was significantly increased in vitiligo patients. Only one patient in the vitiligo group reported taking probiotics, which limits our ability to draw conclusions about the relationship between probiotic use and *Bifidobacterium bifidum* levels. The observed increase in *Bifidobacterium bifidum* proportion may be due to an overall decrease in microbial diversity rather than an absolute increase in *Bifidobacterium bifidum*, and caution will be needed to interpret a causal relationship between the increased *Bifidobacterium bifidum* levels and vitiligo based on our current data. Therefore, additional research is required to elucidate the specific role of *Bifidobacterium bifidum* in vitiligo pathogenesis. Moreover, conflicting results have been reported regarding the abundance of *Bifidobacterium* in patients with various autoimmune diseases. In a recent paper, *Bifidobacterium* and *Lactobacillus* has been found to be increased in active inflammatory bowel disease and Behcet’s disease [22,28].

*Tolypothrix* sp. PCC 7910, *Helicobacter suis*, *Paenibacillus xylanexedens*, *Vibrio aquimaris*, and *Mycolicibacterium alvei* were significantly increased in vitiligo patients, although they occupied little abundance. *Tolypothrix* is a type of filamentous cyanobacteria that produces a diverse range of metabolites particularly in neurodegenerative diseases through mechanisms like protein misincorporation or mitochondrial dysfunction [29]. *Helicobacter suis* is associated with chronic gastritis, peptic ulcer disease, gastric mucosa-associated lymphoid tissue lymphoma, and decreased daily weight gain [30,31]. *Helicobacter suis* infection changed the mucin composition and glycosylation in animal studies [30], implying that Helicobacter species could inhibit mucus-based defenses. *Paenibacillus xylanexedens*, a universal bacteria that degrades xylan [18], has been previously classified as Bacillus groups by 16s rRNA sequencing. In vitro studies and animal studies showed its ability to suppress the number of the *Escherichia coli*; however, its role in humans is not known [32].

The reduction in certain bacterial populations that were predominant in healthy individuals could be the result of the increase in the above dominant species, or it may be the primary cause of the disease, which requires further validation. The decreased abundance of species within Proteobacteriasuch as *Enterobacter bugandensis* and *Neisseria flavescens*, which contribute to a healthy gut microbial system, suggests a compromised gut microbial environment that may fail to maintain proper immune homeostasis.

The strong enrichment of O-glycan biosynthesis pathways in vitiligo patients could suggest several things. Certain enriched microbes in vitiligo patients including bifidobacterium may break down O-glycans and use them for energy and carbon. Secondly, O-glycans are a key component of mucus in the intestinal tract that interacts with gut bacteria, implying that potential alterations in protein glycosylation could affect cellular recognition and immune responses [33]. Moreover, these glycan chains act as self-associated molecular patterns, storing crucial biological information for the immune system. Altered IgG glycosylation pattern was discovered in rheumatoid arthritis patients [34]. In vivo study suggested that modulating IgG sialylation could attenuate autoimmune diseases in mouse models and be a potential new strategy for the treatment of autoimmune diseases [35]. Further analysis of O-glycosylation repertoire in vitiligo patients will be necessary to confirm our finding. The enrichment of endocrine resistance pathways in vitiligo patients may indicate that certain bacteria promoting endocrine resistance to hormones in vitiligo patients, suggesting a potential hormonal component in disease progression.

On the other hand, the riboflavin metabolism and bacterial chemotaxis pathways were significantly enriched in healthy controls. Riboflavin, or vitamin B2, is an essential nutrient crucial for various metabolic processes, particularly in mitochondrial function [36]. Riboflavin serves as a precursor for flavin cofactors, which are involved in various processes, including mitochondrial electron transport and fatty acid β-oxidation [37]. It seems relevant with increasing evidence indicating mitochondrial dysfunction in vitiligo patients [38,39]. Inappropriate bacterial chemotaxis could lead to chronic inflammation or missing many pathogenic bacteria lacking this chemotaxis, which could indicate the increased pathogenic or decreased protective bacteria in vitiligo patients.

Our study intended to find microbial factors that cause the progression of vitiligo, focusing on the comparison between active progressing vitiligo patients. This distinctive patient selection provides a unique perspective compared to previous research in the field. Unlike previous studies on vitiligo and gut microbiome dysbiosis, which primarily focused on Western or Chinese populations, our study is the first to investigate Korean vitiligo patients using shotgun metagenomic sequencing (Table A1). Shotgun sequencing offers several advantages over 16S rRNA sequencing for gut microbiome analysis. It provides enhanced detection of bacterial species, increased diversity, and improved prediction of genes [40]. Shotgun sequencing also allows for deeper characterization of microbiome complexity, identifying a larger number of species per sample [41]. It has more power to detect less abundant taxa, which can be biologically meaningful in discriminating between experimental conditions [42].

This study has several limitations. The number of samples was limited, and we utilized Korean healthy control data from the previously published report. Therefore, we could not control many environmental factors such as diet, medications, and family members. Gut microbiome is known to be primarily affected by environmental factors including diet, drugs, family members, and intestinal motility [43]. However, given Korea’s distinct dietary patterns and potential microbial differences, studying Korean patients provides novel insights into population-specific variations in vitiligo-associated gut dysbiosis. Further studies with larger sample size will be required to elucidate the findings in our study.

## 4. Materials and Methods

### 4.1. Materials

Stool samples were collected from 10 Korean adult vitiligo patients who were diagnosed and treated at The Catholic University of Korea, St. Vincent’s Hospital. These samples were collected sequentially from August 2022 to March 2023, with participants aged from their 20s to 50s, including 2 females and 8 males. Each sample had a read length of 151 bp and was labeled in the collection order as VT1 to VT10. Comparative analyses were conducted between these VT samples and those from a healthy adult control group. We selected stool samples from healthy adults who visited Hanyang University Hospital for the healthy control (HC) group [43]. The HC group consisted of individuals in their 30s, 40s, and 50s, including 8 males and 29 females. Regarding medical history, 11.5% of the participants reported having been prescribed antibiotics in the past year, 34.4% reported not visiting a hospital, and 50.8% visited a hospital fewer than three times during the past year. These samples were obtained from the PRJEB33013 project on the European Nucleotide Archive (ENA), and each sample had a read length of 151 bp [44].

### 4.2. Demographic Matching for Comparative Analysis

**Figure A1:**
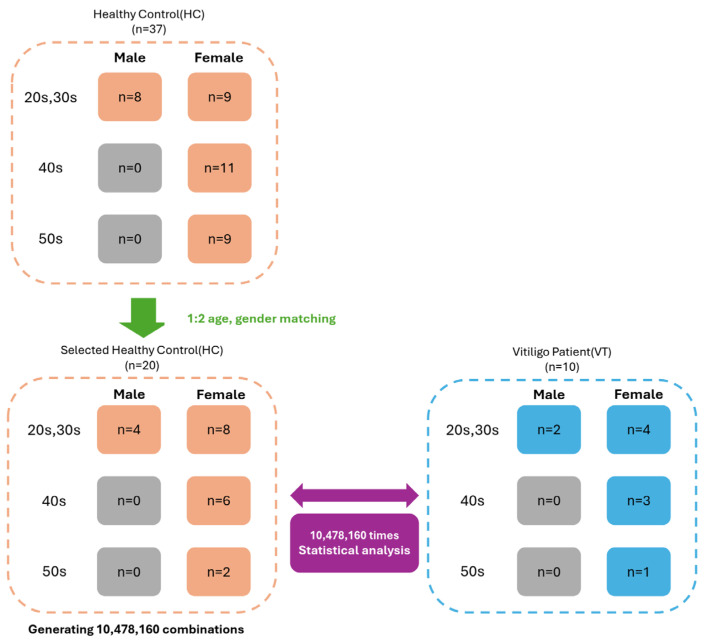
Demographic matching of vitiligo (VT) and healthy control (HC) groups for comparative analysis.

Demographic alignment between the VT and HC groups was achieved through systematic sampling to adjust for age and sex distributions. Twenty samples were systematically extracted from this subset to establish proportional representation corresponding to the VT group’s demographic parameters. This statistical approach yielded 10,478,160 possible sample combinations, where we conducted statistical tests (described in the subsequent section) across all combinations. Additionally, by maintaining proportionality in age and sex while maximizing the number of samples reviewed, we were able to capture trends across the groups more effectively. This sampling approach ensured robust comparative analyses while maintaining demographic homogeneity between cohorts (Figure A1).

### 4.3. Shotgun Sequencing Data Analysis

Shotgun sequencing was conducted to classify each read into its respective taxon, thereby estimating the relative abundance of microbiome taxa and obtaining metabolic pathway data. Raw data for the VT and HC groups were provided in fastq file format, and initial quality control was performed using Sickle (ver. 1.33) [45]. This tool removes low-quality reads, excluding those with quality scores below 20 or lengths under 100 bp to ensure data reliability. Since this is whole-genome sequencing without an amplification process, a high presence of host genome sequences was anticipated. To address this, BWA-MEM2 (ver. 2.2.1) was used to retain only the reads that did not map to the human genome [46].

### 4.4. Taxonomic Classification

For taxonomic classification, Kraken2 was employed. Kraken2 is a highly efficient and accurate tool based on k-mer analysis, which classifies reads to specific taxa. Reads were divided into 35-mers, and each k-mer was compared to the NCBI RefSeq database we constructed, which included bacterial, archaeal, viral, and fungal genomes [47,48]. Kraken2 calculates the weighted sum of k-mers to assign each read to the most suitable taxon, allowing us to determine the relative abundance of microbial taxa.

### 4.5. Calculation of Diversity Indices

Next, we calculated alpha diversity and beta diversity based on Kraken2 results. Alpha diversity is a measure of microbial diversity within each sample, reflecting both species’ richness and evenness. For alpha diversity analysis, we used indices such as the Shannon index and Simpson index [49]. Beta diversity assesses the differences in microbial community composition between the VT and HC groups by comparing microbial communities across samples. For beta diversity, we used non-metric multidimensional scaling (NMDS) to represent rank-based dissimilarities in community structure [50]. NMDS focuses on the rank order of sample distances, allowing for a flexible, non-linear representation of differences between samples based on community composition. This method enabled us to highlight differences in microbial community structure between the VT and HC groups. Results from the beta diversity analysis were visualized using NMDS plots, allowing for a clear comparison of microbial community differences between the groups.

### 4.6. Estimation of Metabolic Pathway Enrichment

The HMP Unified Metabolic Analysis Network (HUMANN3.0) was used to analyze the metabolic functions of the microbial communities. HUMANN3.0 maps shotgun sequencing reads to known gene families and links these to metabolic pathways. Using information from the taxonomic classification, reads were assigned based on amino acid sequences to specific pathways by referencing protein information and annotations in the UniRef database [51,52]. This allowed us to calculate the relative abundance of genes and pathways within the microbial communities. Additionally, HUMANN results were further processed to convert metabolic data into Kyoto Encyclopedia of Genes and Genomes (KEGG) pathways for downstream analysis, enabling a more comprehensive exploration of metabolic functions within the microbial communities.

### 4.7. Statistical Analysis

In the demographic matching process, 20 out of 37 HC samples were selected for comparison with the VT group using the Wilcoxon rank-sum test. Comparison of taxonomic, metabolic, and alpha diversity between the two groups were conducted by Wilcoxon rank-sum test. All analyses were conducted using the R (ver. 4.3.3).

## 5. Conclusions

Overall, these findings indicate that the VT group has a gut microbial imbalance, characterized by reduced diversity and greater variability in microbial composition compared to the HC group. This pattern suggests that the microbiome in the VT group is less resilient and may lack the stability or homeostasis observed in the HC group. These contribute to our understanding of the gut–skin axis in vitiligo and suggest that gut microbiota modulation could be a potential therapeutic target. Future studies should investigate whether restoration of these altered microbial communities could help in disease management or prevention.

## Data Availability

The raw sequencing data generated in this study have been deposited in the NCBI Sequence Read Archive (SRA) under BioProject accession number PRJNA1222148. The data are publicly available.

## Appendix A

**Table A1:** Summary of clinical studies about gut microbiome in vitiligo patients.

Author and Year	Country	Methodology	Sample Size	Alpha Diversity Compared to Controls	Beta Diversity	Main Findings	Pathway Analysis	Metabolome Data
Bzioueche et al., 2021 [8]	France	16S rRNA	10 vitiligo, 10 controls	Reduced	Significantly altered microbial composition	Reduced Bacteroides	Not available	Not available
Kumar et al., 2024 [53]	India	16S rRNA	22 vitiligo, 10 controls	Reduced	Significantly altered microbial composition	Reduced SCFA-producing taxa; increased gut mucosal degradation genes	Not available	SCFA reduction
Ni et al., 2020 [21]	China	16S rRNA	30 vitiligo, 30 controls	Reduced	Significantly altered microbial composition	Decreased Bacteroidetes to Firmicutes ratio; increased Corynebacterium and Psychrobacter	Not available	23 altered metabolites, including taurochenodeoxycholate
Wu et al., 2023 [54]	China	16S rRNA	32 vitiligo, 27 controls	Not available	Significantly altered microbial composition	Increased Firmicutes-to-Bacteroidota ratio in the young adult vitiligo patients; increased Megamonas, Bifidobacterium and Psychrobacter	Paraxanthine, caffeine, and xanthosine from caffeine metabolism pathway downregulated in young vitiligo	1,7-dimethyl uric acid reduction
Luan et al., 2023 [7]	China	Shotgun	25 vitiligo, 25 healthy control	Reduced	No significant difference	Phylum: increased Bacillota; decreased Bacteroidota species: reduced Staphylococcus thermophiles; increased Bacteroides fragilis	NOD-like receptor signaling pathway enriched in vitiligo	Cysteine degradation down-regulated, and galactose degradation up-regulated
Our study	South Korea	Shotgun	10 active spreading vitiligo, 20 controls	Reduced	Significantly altered microbial composition	Phylum: Actinomycetota and Bacteroidota dominance in vitiligo Species: Feacalibacterium prausnizii, Feacalibacterium duncanieae, and Megamonas funiformis were reduced and Bifidobacterium bifidum was enriched	O-glycan biosynthesis enriched in vitiligo	Not available
